# Socio-demographic and behavioural correlates of oral hygiene status and oral health related quality of life, the Limpopo - Arusha school health project (LASH): A cross-sectional study

**DOI:** 10.1186/1471-2431-10-87

**Published:** 2010-11-30

**Authors:** Hawa S Mbawalla, Joyce R Masalu, Anne N Åstrøm

**Affiliations:** 1 Department of Clinical Dentistry, Community Dentistry, University of Bergen, Bergen, Norway; 2 Centre for International Health, University of Bergen, Bergen, Norway; 3 Department of Preventive and Community Dentistry, School of Dentistry, Muhimbili University of Health and Allied Sciences, Dar Es Salaam, Tanzania

## Abstract

**Background:**

Promoting oral health of adolescents is important for improvement of oral health globally. This study used baseline-data from LASH-project targeting secondary students to; 1) assess frequency of poor oral hygiene status and oral impacts on daily performances, OIDP, by socio-demographic and behavioural characteristics, 2) examine whether socio-economic and behavioural correlates of oral hygiene status and OIDP differed by gender and 3) examine whether socio-demographic disparity in oral health was explained by oral health-related behaviours.

**Methods:**

Cross-sectional study was conducted in 2009 using one-stage cluster sampling design. Total of 2412 students (mean age 15.2 yr) completed self-administered questionnaires, whereas 1077 (mean age 14.9 yr) underwent dental-examination. Bivariate analyses were conducted using cross-tabulations and chi-square statistics. Multiple variable analyses were conducted using stepwise standardized logistic regression (SLR) with odds ratios and 95% Confidence intervals (CI).

**Results:**

44.8% presented with fair to poor OHIS and 48.2% reported any OIDP. Older students, those from low socio-economic status families, had parents who couldn't afford dental care and had low educational-level reported oral impacts, poor oral hygiene, irregular toothbrushing, less dental attendance and fewer intakes of sugar-sweetened drinks more frequently than their counterparts. Stepwise logistic regression revealed that reporting any OIDP was independently associated with; older age-groups, parents do not afford dental care, smoking experience, no dental visits and fewer intakes of sugar-sweetened soft drinks. Behavioural factors accounted partly for association between low family SES and OIDP. Low family SES, no dental attendance and smoking experience were most important in males. Low family SES and fewer intakes of sugar-sweetened soft drinks were the most important correlates in females.

Socio-behavioural factors associated with higher odds ratios for poor OHIS were; older age, belonging to the poorest household category and having parents who did not afford dental care across both genders.

**Conclusion:**

Disparities in oral hygiene status and OIDP existed in relation to age, affording dental care, smoking and intake of sugar sweetened soft drinks. Gender differences should be considered in intervention studies, and modifiable behaviours have some relevance in reducing social disparity in oral health.

## Background

Promoting oral health of adolescents through health promoting schools has been prioritized by the World Health Organization (WHO) for the improvement of oral health globally [1]. Adolescents are in particular need for oral health promoting programs [2]. Poor oral hygiene in terms of increasing accumulation of plaque and calculus with increasing age have been reported among children and adolescents in both developed- and developing countries [2,3]. This situation might lead to periodontal problems later in adult life. In Tanzania, the Ministry of Health Policy guidelines have outlined periodontal problems to account for 80% of all oral diseases in the population [4]. Poor oral hygiene in the general Tanzanian population aged 15 years and above is very common (65-99%) with prevalence of gingivitis ranging from 80-90% [5,6]. According to Kerosuo et al [7], a substantial proportion of school students aged 12-18 years and, girls less seriously than boys, presents with sign of gingivitis. In contrast, Kikwilu [8] found a low prevalence of gingivitis and good oral hygiene status among school children in Morogoro.

Appropriate use of inter-dental measures, fluorides, dental services and tooth brushing, restricted frequency sugar intake and avoidance of tobacco consumption contributes to the prevention and control of oral diseases [1]. A recently published national report considering 13-15 years old Tanzanian adolescents showed that about 90% reported daily tooth brushing, whereas the prevalence of adolescents confirming daily intake of sugar products remained at a moderate level [9,10]. Studies have yielded lifetime prevalence rates of tobacco use, ranging from 0.4% to 12% in female- and male adolescents, respectively [9]. Other studies from East Africa focusing adolescents and young adults have reported similar results with respect to oral health enhancing- and oral health detrimental behaviours [11,12].

Untreated oral diseases might lead to dental pain, dysfunction and problems with daily activities [11,13]. To date, oral health related quality of life, OHRQoL, pertaining, to the child- and adolescent populations of Sub-Saharan Africa have been given little attention in the literature. Few studies have assessed the socio-behavioural distribution of OHRQoL and its relationship with clinical indicators of oral hygiene status has yet to be investigated in younger age groups. Instruments are now available for measuring OHRQoL in school-aged children. The Child-OIDP was developed and tested among Thai schoolchildren aged 11-12 yr [14]. It has been found to be a reliable and valid instrument when applied for instance to children and adolescents in Tanzania, France and UK [11,13,15,16].

Socio-economic status has a profound effect on health- and health behaviours [17]. However, inequality in health and oral health has not been focused to the same extent in adolescents as in adults [18,19]. Evidently, the lower the material standard of living as measured by income, social class and social network- and support, the worse the level of oral health, whatever the measures used, being they clinical or self-reported oral health indicators [17]. The World Health Organization (WHO) International Collaborative studies (ICS-I or II), have demonstrated a social gradient in adolescents' caries experience and periodontal status across high-and low income countries and various oral health care service systems [20]. Moreover, social disparities in adolescents' oral health behaviours have been demonstrated in developing countries and elsewhere, with oral health detrimental behaviours being most common in subjects of lower socio-economic status [20]. In Tanzania, previous studies have not given any clear-cut conclusion regarding the relationship between social status and indicators for oral health among children and adolescents.

Owing to scarce resources within the Tanzanian health care sector, it is important to select preventive strategies requiring few resources. It is evident, that oral health interventions through school can improve oral health and oral health related behaviour among adolescents [21]. Youth is believed to be an important period for learning and maintaining health related activities that may carry over into adulthood [22]. Although the Tanzanian oral health policy gives priority to children and adolescents as target groups for health care services, the oral health status and associated life style patterns of this age group are not well documented.

This study uses baseline data from a cluster randomized trial, integrating oral health into a health promoting school programme (LASH), to describe patterns of oral health status and oral health behaviours among secondary school students in Arusha, northern Tanzania. The aims were; 1) to assess the frequency of poor oral hygiene status and oral impacts on daily performances, OIDP, by socio-demographic- and behavioural indicators, 2) to examine whether socio-economic and behavioural correlates of oral hygiene status and OIDP differed by gender and 3) to examine whether socio-demographic disparity in oral health outcomes was explained by oral health behaviours. It was hypothesized that, socio-demographic factors influence oral health outcomes directly or indirectly through oral health related behaviours.

## Methods

### Sampling procedure

A cross sectional study was performed in Arusha, northern Tanzania, focusing secondary school students. In this study area, fluoride concentration in drinking water has been estimated to amount to 3.6 mg fluoride/l drinking water and dental fluorosis is recognized to be endemic [23]. A total of 59 public secondary schools were listed of which 31 schools fulfilled the inclusion criteria of being public schools and having student enrolment of more than 200 students. A sample size of 2000 students was estimated to be satisfactory; assuming that the percentage of students expected to have oral impacts on daily performance was 30%, using an absolute precision (d) of 0.02 and 95% confidence interval (CI) [24]. A one staged stratified cluster design was utilized with secondary school as the primary sampling unit. Secondary schools (n = 31) in the Arusha municipality, Arusha and Meru council were stratified into rural and urban schools, the latter being those within 10 km from Arusha town centre. A total of 11 urban schools (N = 7533 in form I and II) and 20 rural schools (N = 9141 in form I and II) constituted the sampling frame. In the first stage, 10 schools were selected by simple random sampling from rural (k = 10/20) and urban (k = 10/11) schools using an unequal sampling fraction. All available students in Form I and II in the selected urban (1487 out of a total of 4933) and rural (1501 out of a total of 3338) schools were invited to participate in the study. Totals of 1163 and 1249 students in urban and rural schools were subsequently included into the study. The total participation rate was 80.7%. This selection procedure provided a non-self weighted sample of secondary school students in the area. A total of 1077 out of 1331 (participation rate 80.9%) participants enrolled in a random sub sample of 10 schools (5 urban and 5 rural) consented to undergo a full mouth clinical oral examination. The clinical oral examination was conducted in three consecutive days starting from the same day as the main questionnaire survey. Reasons for non-participation in the clinical examination were mainly due to loss of identification numbers for matching purposes, absence from school on the day of examination and dental fear. Parents and students gave written informed consent to participate both in the main questionnaire survey and the clinical examination. Permission to conduct the study was granted by the school authorities and ministries of Education and Health. Ethical clearance was obtained from Muhimbili University of Health and Allied Sciences (MUHAS) and from the National Institute for Medical Research (NIMR) in Tanzania. Ethical approval and research clearance were obtained from the National Committees for research Ethics in Norway and from the Norwegian Social science Data Service.

### Questionnaire

The questionnaire, including 165 questions, was initially constructed in English and translated into Kiswahili, the national language and subsequently back-translated into English by independent translators qualified in English and Kiswahili [Additional file 1]. Following a pilot test, some modifications in terms of clarification and simplification of wording were done. The questionnaires were completed by students in classroom setting under supervision by trained research assistants. *Socio-demographic factors *were assessed in terms of age, gender, place of residence, father's and mother's education, household socio-economic status (perceived affluence of my household) and household wealth index. *Household wealth index *was assessed according to a standard approach in equity analysis [25]. Durable household assets indicative of family wealth (i.e. bicycle, motorcycle, car, TV) were recorded as (1) "available and in working condition" or (0) "not available and/or not in working condition." These assets were analyzed using principal components analysis. The first component resulting from this analysis was used to categorize households into four approximate quartiles of wealth ranging from the 1^st ^poorest quartile to the least poor 4^th ^quartile. *Oral health related behaviours *were assessed in terms of tooth brushing, (e.g. how "frequently do you brush your teeth") dental attendance within the past 2 years, smoking and intake of sugar sweetened soft drinks. Socio-demographic and behavioural characteristics and the number of subjects according to categories are summarized in Table 1. *Oral health related quality of life *was measured using a Kiswahili version [11,13] of the eight item Child OIDP inventory (e.g. During the previous 3 months how often have problems with your teeth and mouth caused you any difficulty with; eating, speaking, cleaning teeth, smiling, sleeping, emotional balance, study and social contact). Each item was scored 0-3 where (0) never, (1) once or twice a month, (2) once or twice a week, (3) every day/nearly every day. A Child-OIDP simple count (SC) score (range 0-8) was constructed by summing the dichotomized frequency items of (1) affected and (0) not affected. Internal consistency reliability (standardized item alpha) of 0.85 agrees with those obtained previously in Tanzania [11,13]. The inter item correlations ranged from 0.31 (difficulty with carrying out major school work or social role and difficulty eating and enjoying food) to 0.55 (difficulty with sleeping and relaxing and difficulty with speaking and pronouncing clearly).

**Table 1:** Frequency distribution of socio-economic and oral health-related behaviours in the main survey and clinical sub-study

*Variable*	*Main questionnaire survey (N = 2412)*	*Clinical sub-study (N = 1077)*
**Age**	% (n)	% (n)
12-15 years	60.6 (1395)	69.8 (752)
16-21 years	39.4 (907)	30.2 (325)
**Sex**		
Male	47.9 (1154)	46,6 (502)
Female	52.1 (1256)	53,4 (575)
**Residence**		
Urban	48.2 (1163)	49.1 (529)
Rural	51.8 (1249)	50.9 (548)
**Mother's education**:		
Low (primary school and below)	65.0 (1247)	68.7 (590)
High (secondary school and above)	35.0 (672)	31.3 (269)
**Father's education**:		
Low (primary school and below)	54.7 (938)	57.7 (445)
High (secondary school and above)	45.3 (778)	42.3 (326)
**Wealth index**:		
1^st ^quartile (Most poor)	22.9 (517)	23.1 (233)
2^nd ^quartile	29.2 (661)	33.7 (340)
3^rd ^quartile	23.0 (521)	21.5 (217)
4^th ^quartile (Least poor)	24.9 (562)	23.1 (219)
**Family SES**		
High	76.5 (1827)	77.5 (825)
Moderate/Low	23.5 (560)	22.5 (239)
**Tooth brushing**		
Not regularly	23.5 (562)	25.5 (273)
Regularly	76.5 (1833)	74.5 (796)
**Parents afford dental care**		
Yes	48.1 (1143)	49.9 (530)
No	51.9 (1234)	50.1 (532)
**Dental visit past 2 years**		
Yes	12.6 (299)	11.3 (120)
No	87.4 (2081)	88.7 (943)
**Sugar-sweetened soft drinks intake**		
Never	46.8 (1116)	45.8 (485)
At least on weekly basis	53.2 (1267)	54.2 (575)
**Tried smoking**		
Yes	5.8 (138)	5.6 (60
No	94.2 (2261)	94.4 (1009)
**Having Oral impact on Daily performance (OIDP)**		
OIDP = 0 (no impact)	51.8 (1204)	49.3 (509)
OIDP > 0 (has impacts	48.2 (1122)	50.7 (524)
**Oral hygiene status**		
Good oral hygiene (OHI-S ≤ 1)		55.2 (594)
Poor oral hygiene (OHI-S > 1)		44.8 (483)

(The total numbers in the different categories do not add up to 2,412 in the main questionnaire survey and to 1,077 in the clinical sub study due to missing values)

### Oral clinical examination

Clinical oral examination was carried out by one trained and calibrated dentist (HSM) assisted by dental assistant for recording the results. Cotton rolls were used to control saliva. Plaque and calculus were assessed under field conditions with adolescents sitting in a regular (non-dental) chair, using natural light, probes and mouth mirror. Oral hygiene was assessed using the Simplified Oral Hygiene Index (OHIS) recognized to be a useful index for evaluation of dental health education in public school systems [26]. Plaque was assessed on 6 index teeth in terms of (0) no debris present, (1) soft debris covering more than one third of the tooth surface, (2) soft debris covering more than one third but not more than two thirds of the tooth surface and (3) soft debris covering more than two thirds of the tooth surface. Calculus was assessed on 6 index teeth and recorded as (0) no calculus present, (1) supra-gingival calculus covering not more than one third of the tooth surface, (2) supra-gingival calculus covering more than one third but not more than two thirds of the tooth surface, (3) supra-gingival calculus covering more than two thirds of the tooth surface. For each individual the debris- and calculus scores of each index tooth were totalled and divided by the number of teeth assessed (range 0-3). The oral hygiene index (OHIS) was constructed by summarizing the debris and calculus scores (range 0-6). For analysis, OHIS scores were dichotomized into 0 = good oral hygiene (OHIS ≤ 1) and 1 = poor oral hygiene (OHIS > 1).

### Statistical analysis

Statistical Package for Social Sciences (SPSS) version 15.0 was used for data analysis. Cluster effect was adjusted for using STATA 10.0. Bivariate analyses were conducted using cross-tabulations and chi-square statistics. Multiple variable analyses were conducted using stepwise standardized logistic regression (SLR) with odds ratios and 95% Confidence intervals (CI). The logistic regression analyses were guided by Petersen's [20] risk factor model for oral diseases, suggesting that socio-environmental factors influence behavioural-and attitudinal factors, which again impact on clinical- and subjective oral health outcomes. To examine whether oral health related behaviours accounted for socio-demographic disparities in oral health status, the approach suggested by Baron and Kenny was adopted [27]. Reduction in ORs for the socio-demographic variables from step I to step II after having included oral health behaviours into the model, was interpreted as evidence of mediation of effects, given that socio-demographic characteristics varied systematically with oral health outcomes and oral health behaviours and that the relationship between oral health outcomes and oral health behaviours were statistically significant.

## Results

### Sample profiles

A total of 2412 out of 2988 eligible secondary school students completed structured questionnaires at school (mean age 15.3 years SD 1.3, response rate 80.7%) and 47.9% were boys. A total of 1077 of 1331 eligible students underwent a full mouth clinical examination providing a response rate of 80.9%, (mean age 14.98 years SD 1.4) and 46.6% were boys. Table 1 gives the percentage distribution of participants' socio-demographics, and oral health related behaviours in the main study group and the clinical sub sample. To assess whether the clinical sub sample (n = 1077) was representative of the study group as a whole a comparison was made with the 1335 students who participated in the questionnaire survey, only. Statistically significant differences were observed between the two samples as the questionnaire only participants had more frequently mothers with high education (31.3% versus 38.0%, p < 0.05), fathers with high education (47.8% versus 42.3%, p < 0.05) and belonged most frequently to the least poor category of the wealth index (.27.4% versus 21.7%, p < 0.05). When a reanalysis was performed with questionnaire variables considering the restricted sample size of 1077 participants only, the findings presented in this paper was essentially unchanged.

### Test retest reliability

Duplicate clinical examination including 25 randomly selected students gave Kappa statistics of 0.783 for calculus score and 0.669 for OHIS score [28].

### Socio-demographic distribution of oral health related behaviours

As shown in Table 1, the majority in both samples brushed their teeth on a daily basis (76.5% and 74.5%) and had not visited a dentist during the past 2 years (87.4% and 88.7%). Only about 5% reported that they had tried cigarettes and about 50% reported intake of sugar sweetened soft drinks on a weekly basis. Pupils with highly educated mothers, younger students, females, those who belonged to the least poor wealth category and high socio-economic status, SES, families presented with intake of sugar sweetened soft drinks more frequently than their counterparts in the opposite groups (p < 0.05) [Additional file 2]. Urban residents, those having parents with high education, belonging to the least poor wealth category and having high SES family status performed regular tooth brushing and dental attendance more frequently than their counterparts in the opposite groups. Finally, older students and males reported smoking more frequently than younger students and females.

### Oral hygiene status and OIDP by socio-behavioural characteristics

Totals of 44.8% had fair to poor oral hygiene (OHIS > 1) whereas 81.1%, 74% and 33% had at least one tooth with plaque, calculus and bleeding, respectively. The mean OHIS score was 1.1, SD 0.8 range (0-4, good-bad) corresponding to a clinical level of fairly good oral hygiene. Totals of 48.2% (in the main sample) and 50.7% (clinical sub sample) reported at least one oral impact on daily performances (OIDP > 0). The most frequently reported impacts were eating problems (36.8%) and problems tooth cleaning (28.9%), whereas the least frequently reported impacts were problems speaking (14.5%) and problems school work (9.9%). Totals of 45.4% versus 58.0% of students having good (OHIS ≤ 1) and poor (OHIS > 1) oral hygiene reported any oral impact on daily performances. Table 2 depicts the overall differences in frequency of students having poor oral hygiene (OHIS > 1) and any oral impact (OIDP > 0) by socio-demographic and behavioural characteristics. As shown, the frequency of oral impacts were higher in older than younger age groups, higher in subjects having father and mother with low education, higher in subjects from low SES families and in those having parents that could not afford dental care. The frequency of having any oral impacts also increased significantly in relation to decreased level of tooth brushing, decreased intake of sugar sweetened soft drinks, increased dental visiting and increased smoking experience. The frequency of having poor oral hygiene increased significantly in relation to increased age, being a male, having father with lower level of education, being in the most poor category of the household category and having parents that could not afford dental care index and in relation to not performing regular tooth brushing (p < 0.05).

**Table 2:** Distribution of Oral hygiene status and OIDP according to socio-demographic and oral health related behaviours

Variable	OHI-S > 1	OIDP > 0
	**% (n) **^**‡**^	**% (n) **^**‡**^
**Age**		
12-15 yr	40.3 (255)	45.1 (607)
16-21 yr	50.3 (197)**	53.3 (466)**
**Sex**		
Male	51.2 (257)	49.8 (551)
Female	39.3 (226)**	46.8 (570)
**Place of residence**		
Urban	43.3 (229)	48.4 (545)
Rural	46.4 (254)	48.0 (577)
**Mother's education**		
Low	47.1 (278)	51.2 (615)
High	38.7 (104)*	44.2 (292)*
**Father's education**		
Low	50.6 (225)	51.8 (466)
High	39.3 (128)**	44.2 (332)**
**Wealth index**		
1^st ^quartile (Most poor)	52.8 (123)	52.2 (262)
2^nd ^quartile	47.4 (161)	48.4 (306)
3^rd ^quartile	44.7 (97)	49.7 (251)
4^th ^quartile (least poor)	32.9 (72)**	43.9 (239)
**Parents afford care**		
Yes	40.0 (212)	41.1 (456)
No	49.6 (264)**	54.7 (649)**
**Family SES**		
High	42.7 (352)	44.8 (791)
Moderate/Low	52.7 (126)	59.6 (319)**
**Tooth brushing**		
Not regularly	51.6 (141)	54.5 (294)
Regularly	42.6 (339)*	46.4 (836)*
**Ever tried smoking**		
No	41.7 (25)	47.7 (1041)
Yes	45.0 (454)	57.9 (77)*
**Dental visit last 2 years**		
Yes	38.3 (46)	56.1 (162)
No	45.5 (429)	47.2 (949)*
**Sugar sweetened soft drink intake**		
Never	48.0 (233)	53.4 (574)
At least on weekly basis	42.3 (243)	43.7 8546)**

**p < 0.001

*p < 0.05

^‡^Frequency of those having OHI-S score >1 and OIDP scores >0, respectively.

All socio-demographic and behavioural variables that were statistically significantly associated with OIDP and OHIS in unadjusted analyses (Table 2) were included into stepwise, logistic regression models. Table 3 depicts adjusted ORs and 95% CI for OIDP by socio-demographics and oral health behaviours. Age, mother's education, father's education and family SES were entered in the first step, providing a model fit of Nagelkerke's R^2 ^= 0.043, Model Chi-Square 45.87 df = 6 p < 0.001. Age, parents' affording dental care and family SES were statistically significant correlates of OIDP in the first step of the model. Entering behavioural variables in the second step, improved the fit of the model to Nagelkerke's R^2 ^= 0.064, Model chi square = 68.81, df = 10, p < 0.001. In the final model, affording dental care and age were the only socio-demographic variables that maintained statistical significance whereas family SES did not. Older students, parents who did not afford dental care and smokers were more likely to report impacts whereas non dental attendees and those who consumed sugar sweetened soft drinks were less likely to report oral impacts as compared with their counterparts in the opposite groups. Statistically significant two way interactions were identified for gender × parents' affording dental care (p < 0.05) and gender × dental attendance (p < 0.05). Stratified logistic regression analyses revealed that parent affording dental care (OR = 1.6, 95% CI 1.2-2.4), dental attendance (OR = 0.4, 95% CI 0.3-0.8) and smoking (OR = 2.6, 95% CI 1.4-5.1) were significant correlates of OIDP in males, whereas family SES (OR = 1.7, 95% CI 1.1-2.7), parents affording dental care (OR = 1.6, 95% CI 1.2-2.3) and intake of sugar sweetened soft drinks (OR = 0.7, 95% CI 0.4-0.9) were significant correlates of OIDP in females. As depicted in Table 4, socio-demographics in terms of age, sex, family wealth index and parents' affording dental care were entered in the first step and provided a model fit in terms of Nagelkerke's R^2 ^of 0.058, Model chi square 38.73, df 6, p < 0.001. All socio-demographic variables remained statistically significantly associated with OHIS in the first step of the models. Entering tooth brushing into the second step improved the model fit to Nagelkerke's R^2 ^0.059, Model chi square 39.30, df 8, p < 0.001. In the final model all socio-demographics, except parents' affording dental care, maintained significant associations with the OHIS score. No statistically significant two way interaction was observed with OHIS.

**Table 3:** OIDP regressed on socio-demographic characteristics and oral health behaviours

Variable	Step 1: **Socio-demographics**^**‡**^	Step 2: **Socio-demographics and behavioural factors**^**§**^
	OR (95% CI)	OR (95% CI)
**Age**		
12-15 yr	1	1
16-21 yr	1.2 (1.0-1.5)	1.2 (1.0-1.5)
**Mother's education**:		
Low	1	1
High	0.9 (0.7-1.2)	0.9 (0.7-1.2)
**Father's education**:		
Low	1	1
High	0.9 (0.7-1.2)	0.9 (0.7-1.2)
**Family SES**		
High	1	1
Moderate/Low	1.3(1.0-1.7)	1.2 (0.9-1.6)
**Parents afford dental care**		
yes	1	1
No	1.6 (1.3-2.0)	1.6 (1.3-2.0)
**Tooth brushing**		
Not regularly		1
Regularly		0.8 (0.6-1.1)
**Ever tried smoking**		
No		1
yes		2.0 (1.2-3.4)
**Dental visit past 2 yr**		
Yes		1
No		0.6 (0.4-0.8)
**Sugar sweetened soft drink intake**		
Never		1
At least on weekly basis		0.7 (0.6-0.9)

^‡^Adjusted Odds ratios (ORs) and 95% Confidence interval (CI) for having at least one OIDP according to socio-demographic characteristics

^§^Adjusted Odds ratios (ORs) and 95% Confidence interval (CI) for having at least one OIDP according to Socio-demographics and oral health behaviours.

**Table 4:** Oral hygiene (OHI-S) regressed on socio-demographic factors and oral health behaviours

Variable	Step 1: **Socio-demographics **^**‡**^	Step 2: **Socio-demographics and toothbrushing**^**§**^
	OR (95% CI)	OR (95% CI)
**Age**		
12-15 yr	1	
16-21 yr	1.3 (1.0-1.9)	1.3 (1.0-1.8)
**Sex**		
Male	1	1
Female	0.6 (0.4-0.8)	0.6 (0.4-0.9)
**Father's education**		
Low	1	1
High	0.7 (0.5-1.2)	0.7 (0.5-1.2)
**Family wealth**		
1st quartile (most poor)	1	1
2nd	0.8 (0.6-1.2)	0.8 (0.6-1.2)
3rd	0.7 (0.5-1.2)	0.8 (0.5-1.2)
4th (least poor)	0.5 (0.3-0.9)	0.5 (0.2-0.7)
**Parents afford dental care**		
yes	1	1
No	1.2 (1.0-1.6)	1.2 (0.9-1.6)
**Tooth brushing**		
No		1
Yes		0.9 (0.6-1.3)

^‡ ^Adjusted Odds ratios (OR) and 95% confidence interval (CI) for having poor oral hygiene (OHIS > 1) according to socio-demographic factors

^§^Adjusted Odds ratios (OR) and 95% confidence interval (CI) for having poor oral hygiene (OHIS > 1) according to socio-demographic factors and tooth brushing

## Discussion

This study reported upon the socio-demographic- and behavioural frequency distribution of oral hygiene status and OIDP in a deprived population of adolescents attending secondary schools examined whether the socio-demographic and -behavioural distribution of oral health outcomes differed between males and females and assessed to what extent oral health related behaviours accounted for socio-economic disparity in oral health outcomes. The factors shown to be statistically significantly and independently associated with higher odds ratios of OIDP included; older age groups, low family SES and parents not affording dental care in addition to smoking experience, no dental visits and fewer intake of sugar sweetened soft drinks. The factors associated with higher odds ratios for poor oral hygiene were older age groups, males and belonging to the poorest category of the household index. Using a stratified approach by gender allowed estimation of a wider range of socio-behavioural disparities with respect to the oral health outcomes investigated. Thus, family SES and intake of sugar sweetened soft drinks were more important correlates of OIDP in females than in males, whereas smoking and dental attendance patterns were most pronounced in males. All socio-behavioural correlates of poor oral hygiene status seemed to be equally important in males and females.

Consistent with many previous studies of child- and adolescents populations in sub Saharan Africa, this study revealed a moderate frequency (prevalence/occurrence) of poor oral hygiene status, high rates of daily tooth brushing, low rates of smoking and moderate intake of sugar sweetened soft drinks [9-13]. These figures indicate that there is a room for improving oral self care, diet and access to and utilization of dental services. Moreover, about half of the students (48%-50%) reported experience with any OIDP during the past 3 months. This rate is higher than those reported previously among similar age groups in Tanzania, but lower than the prevalence rate identified among secondary school students in Uganda and other non-industrialized countries [11,13,29,30]. Problems with eating and cleaning teeth were the most commonly reported impairments, a finding which is consistent with those of other populations using both the adult-and child version of the OIDP inventory [11,13,29,31].

In spite of its considerable floor effect indicating that half of the students investigated did not experience oral impacts, the OIDP inventory exhibited sufficient discriminatory properties suggesting that it is suitable for detecting group differences in cross-sectional studies. Observed OIDP differences across socio-demographic- and behavioural factors were statistically significant both in unadjusted and adjusted analyses and across males and females. In general, these findings confirm the social gradient observed in oral health and oral health related behaviours of adolescent-and adult populations, globally [17,20,32]. Notably, students who had seen a dentist during the previous 2 years reported oral impacts more frequently than students who had not visited a dentist. Similar findings have been reported previously, and might be attributed to symptomatic dental attendance patterns rather than an unexpected response to dental treatment [33]. The social gradient in adolescents' sugar consumption was opposite that observed in industrialized countries being highest in the socially affluent groups of young people. This finding is consistent with evidence suggesting that commercialized sugar products have become highly preferred in low income countries, particularly by the higher socio-economic status groups [34]. Students who reported fewer intake of sugar sweetened soft drinks were more likely than their counterparts to report any impact on daily performances. This might be a reflection of their lower socio-economic status. It is also probably that reduced consumption follows oral impacts in terms of problems eating and cleaning, rather than sugar consumption having advantageous consequences for oral health.

The present findings, suggesting a similar social gradient in oral health behaviours and OIDP and the fact that dental attendance, tooth brushing and intake of sugar sweetened soft drinks varied systematically with OIDP, suggests a contribution of oral health behaviours to social disparities in oral health among the students investigated [35-38]. According to the results depicted in Table 3 and 4, this study suggests that individual behavioural factors might lessen social disparity in oral hygiene status and OHRQoL among adolescents but do not seem to remove it completely. Attempts to explain and describe socio-economic differences in oral health status have mainly focused on adults in industrialized countries, with the commonly held view that poor oral health is explained by personal neglect not always being supported [35-38]. Consistent with the findings in this study, a multilevel analysis of adolescents from 33 industrialized countries revealed that behavioural factors accounted partly for the socio-economic differences in self reported health status [19]. These results appear to imply that preventive programs should focus unhealthy behaviours of adolescents in the poorest socio-economic status groups.

The present results should be interpreted in the light of limitations that include a cross-sectional design, use of self-reported measures and the fact that the estimates presented are not weighted using sample weight. Due to its cross sectional design, the present study cannot demonstrate causality and longitudinal studies are needed to identify the direction of the relationships identified. Another weak aspect is the lack of a measure of dental fluorosis being endemic in the present study area and might assumingly impact children's OHRQoL. Structured, self-administered questionnaires as applied in this study have certain limitations with bias due to social desirability, acquiescence and lack of recall being frequently encountered, particularly in younger age groups [39]. In spite of a reportedly optimal tooth brushing frequency that might counteract the deleterious effects of dental plaque and sugary diets, about 80%, 70% and 30% of the study population presented with plaque, calculus and gingival bleeding. This finding do indicate that response inaccuracy due to recall bias and social desirability is a methodological problem that might have confronted the identification of relationships between oral behaviours and oral health outcomes in this study. However, most findings were in accordance with expectations. Moreover, the measures of oral hygiene - and sugar consumption utilized have been applied previously in East Africa [11-13]. The sugar frequency questionnaire applied has been found to be acceptable with respect to classifying adolescents into broad categories of high and low sugar consumption [12].

## Conclusion

Disparities in oral hygiene status and OIDP existed in relation to age, affording dental care, smoking and intake of sugar sweetened soft drinks. Gender differences should be considered in intervention studies, and modifiable behaviours have some relevance in reducing social disparity in oral health.

## Competing interests

The authors declare that they have no competing interests.

## Authors' contributions

HSM: Principle investigator, designed the study, collected data, performed statistical analyses and writing of the manuscript. ANÅ: Main supervisor, designed study, guided the statistical analyses. She has been actively involved in manuscript writing. JRM: Participated in design of study and provided valuable guidance in data collection. All authors read and approved the final manuscript.

## Pre-publication history

The pre-publication history for this paper can be accessed here:

http://www.biomedcentral.com/1471-2431/10/87/prepub

## Supplementary Material

Additional file 1 **Youth health survey Questionnaire**. A self administered questionnaire used for collection of the information regarding students' basic background information and socio-demographics, oral health related behaviors, Oral Impact on Daily performance index as measure of oral quality of life for the students' baseline information. It has questions on: Individual student and his/her family background, Dietary Behaviors, Oral Health, Tobacco Use and Health services utilization among other things. The mentioned questionnaire sections are relevant to the present study, though the questionnaire had 165 questions. Click here for file

Additional file 2 **Table S5: Socio-demographic distribution of oral health related behaviours**. Table showing percent of the students who reported to consume sugar sweetened soft drink weekly, daily tooth brushing, have tried or are smoking and having attended to a dentist in different socio-demographic groups (N = 2412). Click here for file
