# Supplementary material for: Socio-demographic and behavioural correlates of oral hygiene status and oral health related quality of life, the Limpopo - Arusha school health project (LASH): A cross-sectional study
Source: BMC Pediatr. 2010 Nov 30;10:87. doi: 10.1186/1471-2431-10-87 (PMC3001697; doi:10.1186/1471-2431-10-87)
Supplement: Additional file 1 — Youth health survey Questionnaire. A self administered questionnaire used for collection of the information regarding students' basic background information and socio-demographics, oral health related behaviors, Oral Impact on Daily performance index as measure of oral quality of life for the students' baseline information. It has questions on: Individual student and his/her family background, Dietary Behaviors, Oral Health, Tobacco Use and Health services utilization among other things. The mentioned questionnaire sections are relevant to the present study, though the questionnaire had 165 questions. [file 1471-2431-10-87-S1.DOC]

**Student ID.:--------------------- Questionnaire no.:--------**

**Youth Health Survey**

This is a questionnaire that is used to gather information about young people’s health. It is voluntary for you to participate in this study. You can withdraw from the study at any time, and you can also skip questions that you find too personal to answer. Please do not write your name on this questionnaire. All the information you give us will be kept private; nobody will know who filled in this questionnaire. Your teachers, neighbours, family and other learners will not see your answers.

We are trying to find out better ways of improving the health and oral health of young people. Your responses **are of great value** and will help to keep young people in this country healthy. **Please help us by filling in this questionnaire.**

This is not a test and there are no right or wrong answers. PLEASE BE HONEST IN YOUR ANSWERS.

Please take your time and answer carefully. In questions where there are boxes, please check the box next to the answer you want to give. If you have any questions, please raise your hand and ask the project staffs present in the classroom.

The present study is carried out by Muhimbili University of Health and Allied Sciences (MUHAS) and Centre for Educational Development in Health Arusha (CEDHA).

**THANK YOU VERY MUCH FOR YOUR HELP!**

**School Name: ________________________________________**

**Date: ________________________________**

**Question 1-20 are about you and your family background**

1. What is your sex?

1*. Male*

*2. Female*

2. How old are you? ___________________

3. What is your tribe?

1. *Chagga*
2. *Arusha*
3. *Meru*
4. *Masai*
5. *Pare*
6. *Rangi*
7. *Nyiramba*
8. *Other (please specify)__________*

4. What class are you in?

*1. Form I*

*2. Form II*

5. What is your religion?

1. *Roman catholic*
2. *Lutheran*
3. *Islam*
4. *Pentecost*
5. *Other (please specify)* ____________

6. Which of the following languages are spoken at home? Tick all that apply.

1. *English*
2. *Swahili*
3. *Other (please specify)__________*

7. What is your place of residence?

*_________________________*

8. Do you live with both parents?

1. *Yes*
2. *No*

9. If no why?

1. *divorce*
2. *Father died*
3. *Mother died*
4. *Father and mother died*
5. *Not applicable*

10. What is the highest level of education your father has?

1. *No formal education*
2. *Primary education*
3. *Secondary education*
4. *College/university education*
5. *Father died*
6. *I do not know*

11. What is your mother’s the highest level of education?

1. *No formal education*
2. *Primary education*
3. *Secondary education*
4. *College/university education*
5. *Mother died*
6. *I do not know*

12. Do you have any of the following things in your home? Only answer “Yes” if you have them and they work.

i) Television

1. *Yes*
2. *No*

ii) Electricity

1. *Yes*
2. *No*

iii) Bicycle

1. *Yes*
2. *No*

iv) Tap water

1. *Yes*
2. *No*

v ) Motor car

1. *Yes*
2. *No*

*vi) Flush toilet*

1. *Yes*
2. *No*

13. Which of the following best describes your home?

1. *Cemented brick house*
2. *Burnt brick house*
3. *Mud brick house*
4. *mud house*
5. *iron sheet house*
6. *wood house*

14. The roof of your house is made up of……..

1. Iron sheets
2. Grass
3. Tiles
4. Others (mention)

15. How many people sleep in the same room with you at night when you are at home?

_____________

16. Which of the following is true of your home? Please mark the statement that best describes your situation:

1. *Well off*
2. *Moderate economical status*
3. *Low economical status*

17. Have you ever repeated a school year due to failing exams?

1. *Yes*
2. *No*

18. How many days were you absent from school during the last school term?

1. _____________
2. Never been absent

19. Do you think you will complete your schooling up to form four?

1. *Yes*
2. *No*
3. *I don’t know*

20. What do you think you will do when you finish secondary school?

1. *Form v*
2. *Go to trade school (VETA)*
3. *Casual labour*
4. *Start a business*
5. *I don’t know*
6. *Other, specify___________*

**Question 21-29are on Alcohol and drug use**

21. Have you ever tasted alcohol?

1. *Yes*
2. *No*

22 During the past 30 days, on how many days did you have at least one drink containing alcohol?

1. *____________________*
2. *I did not drink alcohol in the last 30 days*

23 During the past 30 days, on the days you drank alcohol, how many drinks did you usually drink per day?

1. *I did not drink alcohol in the last 30 days*
2. *Less than 1 drink*
3. *1 drink*
4. *2 drinks*
5. *3 drinks*
6. *4 drinks*
7. *5 or more drinks*

24 How many of your friends drink alcohol on a regular basis?

1. *None of them*
2. *Some of them*
3. *Most of them*
4. *All of them*
5. *I don’t know*

25 Have you ever tried to use drugs/substances of abuse such as bangi, or cocaine**?**

1. *Yes*
2. *No*

26 During the past 30 days, how many times have you used drugs/substances of abuse such as such as bangi or cocaine?

1. *________________________*
2. *Never*

27What types of drugs have you tried most times?

1. *____________________________*
2. *Never*

28 How many of your friends have tried drugs/substances of abuse such as bangi or cocaine?

1. *None of them*
2. *Some of them*
3. *Most of them*
4. *All of them*
5. *I don’t know*

29 During this school year, were you taught in any of your classes the dangers of drug use?

1. *Yes*
2. *No*

**Questions 30- 48 are on Dietary Behaviours**

30. During the past 30 days, how often did you eat breakfast?

1. *__________________________*
2. *Never*

31. During the past 30 days, how often did you bring lunch to school?

1. *Never*
2. *Rarely*
3. *Sometimes*
4. *Most of the time*
5. *Always*

32. During the past 30 days, how often was breakfast offered to you at school?

1. *Never*
2. *Rarely*
3. *Sometimes*
4. *Most of the time*
5. *Always*

33. During the past 30 days, how often were you hungry at school?

1. *Never*
2. *Only a few times*
3. *1-2 day a week*
4. *3-4 days a week*
5. 5-6 days a week
6. Every day

34. During the past 30 days, how often was a snack offered to you at school?

1. *Never*
2. *Rarely*
3. *Sometimes*
4. *Most of the time*
5. *Always*

*35. In the past 30 days, when you ate food at school, where did the food come from? Please mark all that apply*

1. *I didn’t have any foods at school in the last 30 days*
2. *I did bring food from home*
3. *My school provides food for students*
4. *Families provide foods for students*
5. *Community members provide foods for students*
6. *I bought food at school*

36. During the past 7 days, on how many days did you eat fast foods such as Chips, eggs etc?

1. *Never*
2. *rarely*
3. *sometimes*
4. *most of the times*
5. *everyday*

37. During the past 30 days, how often did you usually eat fruit such as ripe bananas, oranges, pawpaw, mangoes or pineapples?

1. *Never*
2. *Once a day*
3. *2 times a day*
4. *3 times a day*
5. *4 times a day*
6. *5 or more times a day*

38. During the past 30 days, how often did you usually eat vegetables such as amaranth, cassava leaves, pumpkin leaves, cabbage, spinach, occra or carrots?

1. *Never*
2. *Once a day*
3. *2 times a day*
4. *3 times a day*
5. *4 times a day*
6. *5 or more times a day*

39. During the past 30 days, how often do you drink sugar sweetened soft drinks, such as Coke or Mirinda?

1. *I have never*
2. *once every day*
3. *often every day*
4. *often per week*
5. *rarely*

40. During the past 30 days, how often do you eat sweets like chocolate and candy?

1. *Never*
2. *Rarely*
3. *Sometimes*
4. *Most of the time*
5. *Always*

41. During this school year, were you taught in any of your classes about the benefit of eating healthy food?

1. *Yes*
2. *No*

42. (a) “The food that we bought just didn’t last, and we didn’t have money to get more, how often did this happen – almost every month, some months but not every month, or in only one or two months?

1. *Almost every month*
2. *Some months, but not every month*
3. *In only 1 or 2 months*
4. *Never happened*

(b) “We couldn’t afford to eat balanced meals, how often did this happen – almost every month, some months but not every month, or in only one or two months?

1. *Almost every month*
2. *Some months, but not every month*
3. *In only 1 or 2 months*
4. *Never happened*

(c) In the last 12 months did you or adults in your household ever cut the size of your meals or skip meals because there wasn't enough money for food, how often did this happen – almost every month, some months but not every month, or in only one or two months?

1. *Almost every month*
2. *Some months, but not every month*
3. *In only 1 or 2 months*
4. *Never happened*

(d) If your family ever cut the size of meals or skip meals because there wasn’t enough money for schools, how often did this happen – almost every month, some months but not every month, or in only one or two months?

1. *Almost every month*
2. *Some months, but not every month*
3. *In only 1 or 2 months*
4. *Never happened*

43. In the last 12 months, did you ever eat less than you felt you should because there wasn't enough money for food?

1. *Yes*
2. *No*
3. *Don’t know*

44. In the last 12 months, were you ever hungry but didn't eat because there wasn't enough money for food?

1. *Yes*
2. *No*
3. *Don’t know*

45. In the last 12 months, how often did you go hungry because there was not enough food in your home?

1. *Almost every month*
2. *Some months, but not every month*
3. *In only 1 or 2 months*
4. *Never happened*

46. During the past 12 months, have you been weighed and measured?

1. *Yes*
2. *No*

47. How do you describe your weight?

1. *Very underweight*
2. *Slightly underweight*
3. *About the right weight*
4. *Slightly overweight*
5. *Very overweight*

48. Which of the following are you trying to do about your weight?

1. *I am not trying to do anything about my weight*
2. *Lose weight*
3. *Gain weight*
4. *Stay the same weight*

**Questions 49- 60 are on Social network and decision-making**

**49. There are many different ways to make a decision or a choice. In the past 12 months, how often have you talked to the following people for help or help to make a decision?**

(i) Talked to your parents?

1. *Never*
2. *Sometimes*
3. *Often*

(ii) Talked to your best friend?

1. *Never*
2. *Sometimes*
3. *Often*

(iii) Talked to a teacher?

1. *Never*
2. *Sometimes*
3. *Often*

(iv) Talked to religious leader?

1. *Never*
2. *Sometimes*
3. *Often*

(v) Talked to a health person like a doctor or a nurse or a healer?

1. *Never*
2. *Sometimes*
3. *Often*

(vi) Talked to some other adult in your family besides your parent (for example, your grandmother, an uncle, your older sister?)

1. *Never*
2. *Sometimes*
3. *Often*

50. How confident are you that you could say no to drink alcohol when you do not want to drink alcohol?

1. *Very confident*
2. *Somewhat confident*
3. *Not very confident*
4. *Not at all confident*

51. How confident are you that you could say “no” if you were offered a cigarette?

1. *Very confident*
2. *Somewhat confident*
3. *Not very confident*
4. *Not at all confident*

52. How confident are you that you could say “no” if you were offered marijuana?

1. *Very confident*
2. *Somewhat confident*
3. *Not very confident*
4. *Not at all confident*

53. How confident are you that you could avoid a physical fight if someone wanted to start a fight with you?

1. *Very confident*
2. *Somewhat confident*
3. *Not very confident*
4. *Not at all confident*

54. How confident are you that you could say “no” to have sexual intercourse against your will?

1. *Very confident*
2. *Somewhat confident*
3. *Not very confident*
4. *Not at all confident*

55. How confident are you that you could ask a person who is bothering you to stop?

1. *Very confident*
2. *Somewhat confident*
3. *Not very confident*
4. *Not at all confident*

56. How easy or hard is it for you to do physical activities?

1. *Very hard*
2. *Hard*
3. *Easy*
4. *Very easy*
5. *Not sure*

57. How easy or hard is it for you to eat a healthy amount of fruits and vegetables?

1. *Very hard*
2. *Hard*
3. *Easy*
4. *Very easy*
5. *Not sure*

58. How easy or hard is it for you to avoid eating too many sweets?

1. *Very hard*
2. *Hard*
3. *Easy*
4. *Very easy*
5. *Not sure*

59. How easy or hard is it for you to avoid eating too many fried foods?

1. *Very hard*
2. *Hard*
3. *Easy*
4. *Very easy*
5. *Not sure*

60. How easy or hard is it for you to use a condom when having sexual intercourse?

1. *Very hard*
2. *Hard*
3. *Easy*
4. *Very easy*
5. *Not sure*

**Questions 61- 68 are on Hygiene**

61. Is there a source of clean water for drinking at your school?

1. *Yes*
2. *No*

62. During this school year, were you taught in any of your classes how to avoid worm infections?

1. *Yes*
2. *No*

63. During this school year, were you taught in any of your classes where to get treatment for a worm infection?

1. *Yes*
2. *No*

64 During the past 30 days, how often did you use the toilets or latrines at school?

1. *Never*
2. *Rarely*
3. *Sometimes*
4. *Everyday*

65. During the past 30 days, how often did you wash your hands after using the toilet or the latrine

1. *Never*
2. *Rarely*
3. *Sometimes*
4. *Most of the times*
5. *Always*

66. During the past 30 days, how often did you wash your hands before eating?

1. *Never*
2. *Rarely*
3. *Sometimes*
4. *Most of the times*
5. *Always*

67 During the past 30 days, how often did you use soap when washing your hands?

1. *Never*
2. *Rarely*
3. *Sometimes*
4. *Most of the times*
5. *Always*

68. During this school year, were you taught in any of your classes the importance of hand washing?

1. *Yes*
2. *No*

**Questions 69- 80 are on Oral Health**

69. During the past 3 months- how often have problems with your mouth or teeth for example such as mentioned above or other caused you any difficulty with eating and enjoying food?

1. *Never*
2. *Once or twice a month*
3. *Once or twice a week*
4. *Every or nearly every day*

70. During the past 3 months- how often have problems with your mouth or teeth caused you any difficulty with speaking and pronouncing clearly?

1. *Never*
2. *Once or twice a month*
3. *Once or twice a week*
4. *Every or nearly every day*

71. During the past 3 months- how often have problems with your mouth or teeth caused you any difficulty with cleaning teeth?

1. *Never*
2. *Once or twice a month*
3. *Once or twice a week*
4. *Every or nearly every day*

72. During the past 3 months- how often have problems with your mouth or teeth caused you any difficulty with sleeping and relaxing?

1. *Never*
2. *Once or twice a month*
3. *Once or twice a week*
4. *Every or nearly every day*

73. During the past 3 months- how often have problems with your mouth or teeth caused you any difficulty with smiling, laughing and showing teeth without embarrassment?

1. *Never*
2. *Once or twice a month*
3. *Once or twice a week*
4. *Every or nearly every day*

74. During the past 3 months- how often have problems with your mouth or teeth caused you any difficulty with maintaining usual emotional state without being irritable?

1. *Never*
2. *Once or twice a month*
3. *Once or twice a week*
4. *Every or nearly every day*

75. During the past 3 months- how often have problems with your mouth or teeth caused you any difficulty with carrying out major school work or social role?

1. *Never*
2. *Once or twice a month*
3. *Once or twice a week*
4. *Every or nearly every day*

76. During the past 3 months- how often have problems with your mouth or teeth caused you any difficulty with enjoying contact with people?

1. *Never*
2. *Once or twice a month*
3. *Once or twice a week*
4. *Every or nearly every day*

77. How would you describe the health of your teeth and gums?

1. *Very poor*
2. *Poor*
3. *Good*
4. *Very good*

78. How satisfied are you with the appearance of your teeth?

1. *Very satisfied*
2. *Satisfied*
3. *Dissatisfied*
4. *Very dissatisfied*

79. How often do you brush your teeth?

1. *Never*
2. *Rarely*
3. Most of the times
4. Every day
5. I don’t brush my teeth

80. What type of toothpaste do you use ?

1. *_______________________*
2. *Do not use tooth paste*

**Questions 81- 87 are on Mental Health**

81. During this school year, were you taught in any of your classes how to handle stress in healthy ways?

1. *Yes*
2. *No*

82 In general, how do you feel about your life?

1. *I feel very happy*
2. *I feel happy*
3. *I feel not very happy*
4. *I do not feel happy at all*

83 In general, how do you think about yourself?

1. *I am very satisfied*
2. *I am quite satisfied*
3. *I am not very satisfied*
4. *I am not satisfied at all*

84 I often feel sad (depressed) without knowing why.

1. *I strongly agree*
2. *I agree*
3. *I disagree*
4. *I strongly disagree*

85 Sometimes I feel everything is so hopeless, that I do not want to do anything.

1. *I strongly agree*
2. *I agree*
3. *I disagree*
4. *I strongly disagree*

86 Sometimes I have been thinking that my life is not worth living.

1. *I strongly agree*
2. *I agree*
3. *I disagree*
4. *I strongly disagree*

87 During this school year, were you taught in any of your classes how to deal with mental health issues?

1. *Yes*
2. *No*

**Questions 88- 94 are on Physical Activity**

88. During this school year, on how many days did you go to physical education class each week?

1. *Never*
2. *1 day*
3. *2 days*
4. *3 days*
5. *4 days*
6. *5 or more days*

89. Outside school hours, how often do you usually exercise so much that you get out of breath or sweat?

1. *Every day*
2. *4-6 times a week*
3. *2-3 times a week*
4. *Once a week*
5. *Once or twice a month*
6. *Never*

90. During this school year, were you taught in any of your classes the benefits of physical activity?

1. *Yes*
2. *No*

91. How do you normally get to and from school?

1. *Walking*
2. *Cycling*
3. *Bus*
4. *Private car*
5. *Other (specify)___________*

92. If you walk to school, about how long does it take you to walk EACH WAY?

1. *Never walked to school*
2. *Less than 9 minutes each way*
3. *10 to 19 minutes each way*
4. *20 to 29 minutes each way*
5. *30 to 39 minutes each way*
6. *40 to 49 minutes each way*
7. *50 to 59minutes each way*
8. *60 or more minutes each way*

93. During a week how many hours do you spend watching television or videos/DVD?

1. *Less than ½ hour*
2. *½ to 2 hours*
3. *2 ½ to 4 hours*
4. *4 ½ to 6 hours*
5. *More than 6 hours*
6. *I don’t watch television or videos/DVD*

*94. During a week how many hours do you spend on the computer or internet?*

1. *Less than ½ hour*
2. *½ to 2 hours*
3. *2 ½ to 4 hours*
4. *4 ½ to 6 hours*
5. *More than 6 hours*
6. *Never used a computer or internet*

**Questions 95- 108 are on Sexual Behaviors**

95. Have you ever had a girl-/boyfriend?

1. *Yes*
2. *No*

96. Have you ever had more than one girl-/boyfriend at the same time?

1. *Yes*
2. *No*

97 Have you ever had vaginal sexual intercourse? This meaning intimate contact with someone during which the penis enters the vagina (female private parts).

1. *Yes*
2. *No*

98. Have you ever had oral sex? This meaning intimate contact with someone during which penis is in the mouth or mouth to vagina or mouth to anus.

1. *Yes*
2. *No*

99. Have you ever had anal sex? This means sexual intercourse during which the penis enters the anus

1. *Yes*
2. *No*

100. During the past 12 months, how many times did you have sexual intercourse?

1. *__________________________*
2. *I have never had sexual intercourse*

101. Have you ever used a condom during sexual intercourse?

1. *I have never had sexual intercourse*
2. Yes
3. *No*

102. The last time you had sexual intercourse, did you or your partner use any method of birth control, such as withdrawal, rhythm (safe time), birth control pills, or any other method to prevent pregnancy?

1. *I have never had sexual intercourse*
2. *Yes*
3. *No*
4. *I don’t know*

103. During the past 12 months, with how many people have you had sexual intercourse?

1. *I have never had sexual intercourse*
2. *I have had sexual intercourse, but not during the past 12 months*
3. *1 person*
4. *2 people*
5. *3 people*
6. *4 people*
7. *5 people*
8. *6 or more people*

104. How many of your friends have had sexual intercourse?

1. *None of them*
2. *Some of them*
3. *Most of them*
4. *All of them*
5. *I don’t know*

105. During this school year, were you taught in any of your classes how to use a condom?

1. *Yes*
2. *No*

106. During this school year, were you taught in any of your classes about sexuality?

1. *Yes*
2. *No*

107. During this school year, were you taught in any of your classes how to avoid HIV infection or AIDS?

1. *Yes*
2. *No*

108. During this school year, were you taught in any of your classes where to get tested for HIV infection or AIDS?

1. *Yes*
2. *No*

**Questions 109- 115 are on Tobacco Use**

109. Have you ever tried or experimented with cigarette smoking, even one or two puffs?

1. *Yes*
2. *No*

110. During the past 30 days, on how many days did you smoke cigarettes?

1. *_______________________*
2. *Never tried cigarette smoking*

111. During the past 30 days, on how many days did you use any other form of tobacco, such as tobacco roll, snuff, or chew tobacco?

1. *_______________________*
2. *Never tried or experimented cigarette smoking*

112. Has a cigarette company representative ever offered you a free cigarette?

1. *Yes*
2. *No*

.

113. During the past 30 days, did anyone ever refuse to sell you cigarettes because of your age?

1. *Never tried or experimented cigarette smoking*
2. *I did not try to buy cigarettes during the past 30 days*
3. *Yes, someone refused to sell me cigarettes because of my age sell me cigarettes because of my age*
4. *No, my age did not keep me from buying cigarettes*

114. How many of your friends smoke cigarettes on a regular basis?

1. *None of them*
2. *Some of them*
3. *Most of them*
4. *All of them*

115. During this school year, were you taught in any of your classes the dangers of tobacco use?

1. *Yes*
2. *No*

**Questions 116- 124 are on Violence**

116. During the past 12 months, how many times were you physically attacked?

1. *________________________*
2. *Never*

117. During the past 12 months, how many times were you in a physical fight?

1. *________________________*
2. *Never*

118. During the past 30 days, on how many days were you bullied?

1. *________________________*
2. *Never*

119. During the past 30 days, on how many days did you carry a weapon, such as a gun, knife, club etc.?

1. *________________________*
2. *Never*

120. During the past 12 months, how many times were you seriously injured?

1. *________________________*
2. *Never*

121. During the past 12 months, has someone ever threatened to use knife or other weapon against you?

1. *Yes*
2. *No*

122 During the past 12 months, have you ever threatened s a girl-/boyfriend ever threatened to someone with knife or other weapon?

1. *Yes*
2. *No*

123. During the past 12 months, have you been physically forced to have sexual intercourse when you did not want to?

1. *Yes*
2. *No*

124. During this school year, were you taught in any of your classes how to reduce and avoid violence?

1. *Yes*
2. *No*

**Questions 125- 152 are on School environment. Please agree or disagree with the following statements about your school**

125. I like to go to school

1. *Strongly agree*
2. *Agree*
3. *Neither agree or disagree*
4. *Disagree*
5. *Strongly disagree*

126. There are enough toilets or latrines at my school

1. *Strongly agree*
2. *Agree*
3. *Neither agree or disagree*
4. *Disagree*
5. *Strongly disagree*

127. The toilets and latrines at my school are easy to get to

1. *Strongly agree*
2. *Agree*
3. *Neither agree or disagree*
4. *Disagree*
5. *Strongly disagree*

128. There is water to wash my hands after using the toilet at my school

1. *Strongly agree*
2. *Agree*
3. *Neither agree or disagree*
4. *Disagree*
5. *Strongly disagree*

129. There is water to wash my hands before I eat meals or snacks at my school.

1. *Strongly agree*
2. *Agree*
3. *Neither agree or disagree*
4. *Disagree*
5. *Strongly disagree*

130. I have the freedom to express my own meanings and opinions at school.

1. *Strongly agree*
2. *Agree*
3. *Neither agree or disagree*
4. *Disagree*
5. *Strongly disagree*

131. My school is concerned about my health and well-being.

1. *Strongly agree*
2. *Agree*
3. *Neither agree or disagree*
4. *Disagree*
5. *Strongly disagree*

132. Students at my school are involved in planning health education programs for youth.

1. *Strongly agree*
2. *Agree*
3. *Neither agree or disagree*
4. *Disagree*
5. *Strongly disagree*

133. I am encouraged by my school to take part in meetings and help plan youth health activities.

1. *Strongly agree*
2. *Agree*
3. *Neither agree or disagree*
4. *Disagree*
5. *Strongly disagree*

134. My school discourages students from using tobacco

1. *Strongly agree*
2. *Agree*
3. *Neither agree or disagree*
4. *Disagree*
5. *Strongly disagree*

135. My school discourages students from drinking alcohol

1. *Strongly agree*
2. *Agree*
3. *Neither agree or disagree*
4. *Disagree*
5. *Strongly disagree*

136. My school discourages students from using drugs

1. *Strongly agree*
2. *Agree*
3. *Neither agree or disagree*
4. *Disagree*
5. *Strongly disagree*

137. I have chances to play and be active while I am at school

1. *Strongly agree*
2. *Agree*
3. *Neither agree or disagree*
4. *Disagree*
5. *Strongly disagree*

138. I feel safe at school

1. *Strongly agree*
2. *Agree*
3. *Neither agree or disagree*
4. *Disagree*
5. *Strongly disagree*

139. My school expects students to be respectful to each other

1. *Strongly agree*
2. *Agree*
3. *Neither agree or disagree*
4. *Disagree*
5. *Strongly disagree*

140. My school expects that teachers are respectful of students

1. *Strongly agree*
2. *Agree*
3. *Neither agree or disagree*
4. *Disagree*
5. *Strongly disagree*

141. My school cares if students are hungry during the day

1. *Strongly agree*
2. *Agree*
3. *Neither agree or disagree*
4. *Disagree*
5. *Strongly disagree*

142. If I was sick or needed help when I was at school, my teachers would help me

1. *Strongly agree*
2. *Agree*
3. *Neither agree or disagree*
4. *Disagree*
5. *Strongly disagree*

143. Girls are treated the same as boys at my school

1. *Strongly agree*
2. *Agree*
3. *Neither agree or disagree*
4. *Disagree*
5. *Strongly disagree*

144. My school cares about my sexual health

1. *Strongly agree*
2. *Agree*
3. *Neither agree or disagree*
4. *Disagree*
5. *Strongly disagree*

145. During the past 30 days, on how many days did you feel unsafe at school or on your way to and/or from school?

1. *Never*
2. *1 time*
3. *2 or 3 times*
4. *4 or 5 times*
5. *6 or more times*

146. To what extent do people of your age in your village participate in sport and recreation?

1. *To very large extent*
2. *To a large extent*
3. *To some extent*
4. *Not at all*

147. To what extent do people of your age in your village participate in economic activities in the village?

1. *To very large extent*
2. *To a large extent*
3. *To some extent*
4. *Not at all*

148 To what extent do people of your age in your village participate in health promoting activities?

1. *To very large extent*
2. *To a large extent*
3. *To some extent*
4. *Not at all*

149. To what extent do people of your age in your village participate in defense and security of the community?

1. *To very large extent*
2. *To a large extent*
3. *To some extent*
4. *Not at all*

150. To what extent do people of your age in your village participate in leadership and community management?

1. *To very large extent*
2. *To a large extent*
3. *To some extent*
4. *Not at all*

151. People of my age are involved in planning and setting priorities regarding community activities concerning youth?

1. *I strongly agree*
2. *I agree*
3. *I neither agree or disagree*
4. *I disagree*
5. *I strongly disagree*

152. People of my age play an important role in how my community runs?

1. *I strongly agree*
2. *I agree*
3. *I neither agree or disagree*
4. *I disagree*
5. *I strongly disagree*

**Questions 153- 156 are generally about your health**

153. How satisfied are you with your health?

1. *Very satisfied*
2. *Satisfied*
3. *Dissatisfied*
4. *Very dissatisfied*

154. During past 3 months, how often has your health, or how you are feeling, made it difficult for you to get to school?

1. *Never*
2. *Rarely*
3. *One to two times a month*
4. *Once or twice a week*
5. *Every day*

155. During the past 3 months, how often has your health, or how you are feeling, made it difficult for you to spend time with your friends?

1. *Never*
2. *Rarely*
3. *One to two times a month*
4. *Once or twice a week*
5. *Every day*

156. During the past 3 months, how often has your health, or how you are feeling, made it difficult to participate in activities with your family?

1. *Never*
2. *Rarely*
3. *One to two times a month*
4. *Once or twice a week*
5. *Every day*

**Questions 157- 163 are on Health services**

157. During the past 12 months, did you ever consult anyone with an issue regarding your health?

1. *Yes*
2. *No*

158. If you need health care service, how easy would it be for you to obtain such service?

1. *Very easy*
2. *Easy*
3. *Neither easy or difficult*
4. *Difficult*
5. *Very difficult*

159. Can your parents afford to pay for health services?

1. *Yes*
2. *No*
3. *I don’t know*

160. If you need oral health care service, how easy would it be for you to obtain such service?

1. *Very easy*
2. *Easy*
3. *Neither easy or difficult*
4. *Difficult*
5. *Very difficult*

161. If you needed oral health care service, would your parents be able to afford these services?

1. *Yes*
2. *No*

162. During the past 2 years have you attended a dental clinic in order to receive help or treatment?

1. *Yes*
2. *No*

163. Who would you prefer to talk to if you had a health problem (tick of more than one alternative if necessary)?

1. *Someone at a health clinic*
2. *A medical doctor*
3. *Traditional healer*
4. *My mother/female guardian*
5. *My father/male guardian*
6. *A relative*
7. *A teacher*
8. *A friend*
9. *Other (specify)*

*_____________________________*

**After filling this questionnaire please go where your weight and height will be measured**

Weight _______________ Kg

Height _______________ cm

THANK YOU VERY MUCH FOR YOUR TIME AND EFFORT!
