# Supplementary material for: Socio-demographic and behavioural correlates of oral hygiene status and oral health related quality of life, the Limpopo - Arusha school health project (LASH): A cross-sectional study
Source: BMC Pediatr. 2010 Nov 30;10:87. doi: 10.1186/1471-2431-10-87 (PMC3001697; doi:10.1186/1471-2431-10-87)
Supplement: Additional file 2 — Table S5: Socio-demographic distribution of oral health related behaviours. Table showing percent of the students who reported to consume sugar sweetened soft drink weekly, daily tooth brushing, have tried or are smoking and having attended to a dentist in different socio-demographic groups (N = 2412). [file 1471-2431-10-87-S2.DOC]

**Table 5**

**Socio-demographic distribution of oral health related behaviors**

| Variable | Sugar sweetened soft drink intake | Tooth brushing | Smoking | Dental attendance |
| --- | --- | --- | --- | --- |
|  | % (n) | % (n) | % (n) | % (n) |
| **Age** |  |  |  |  |
| 12-15 yr | 57.7 (797) | 77.8 (1077) | 4.4 (61) | 12.7 (175) |
| 16-21 yr | 45.9 (410)** | 75.4 (679) | 7.4 (67)** | 11.3 (101) |
| **Sex** |  |  |  |  |
| Male | 48.2 (550) | 73.0 (834) | 8. 9 (102) | 15.0 (170) |
| Female | 57.6 (715)** | 79.7 (997)** | 2.9 (36)** | 10.3 (128)** |
| **Place of residence** |  |  |  |  |
| Urban | 53.6 (616) | 82.9 (957) | 5.9 (68) | 13.9 (161) |
| Rural | 52.8 (651) | 70.6 (876)** | 5.6 (70) | 11.3 (138)* |
| **Mother’s education:** |  |  |  |  |
| Low | 47.7 (588) | 74.7 (926) | 6.4 (79) | 10.4 (128) |
| High | 63.3 (420)** | 80.1 (534)* | 5.3 (35) | 16.2 (107)** |
| **Father’s education** |  |  |  |  |
| Low | 50.2 (467) | 73.0 (680) | 5.4 (50) | 10.4 (97) |
| High | 61.6 (471)** | 82.3 (637 )** | 6.2 (48) | 16.2 (124)** |
| **Family wealth** |  |  |  |  |
| 1st quartile (most poor) | 38.0 (194) | 69.8 (360) | 5.4 (28) | 9.6 (49) |
| 2nd | 52.9 (343) | 70.8 (462) | 5.0 (33) | 9.7 (63) |
| 3rd | 55.7 (287) | 78.7 (407) | 6.2 (32) | 13.2 (68) |
| 4th (least poor) | 64.2 (358)** | 87.0 (488)** | 6.1 (34) | 17.4 (97)** |
| **House SES** |  |  |  |  |
| High | 60.3 (1090) | 79.0 (1430) | 5.5 (100) | 13.5 (243) |
| Low | 29.3 (162)** | 68.5 (383)** | 6.5 (36) | 9.2 (51)* |

***p< 0.0001;

**p< 0.005;

*p< 0.05
